# Experts’ Failure to Consider the Negative Predictive Power of Symptom Validity Tests

**DOI:** 10.3389/fpsyg.2022.789762

**Published:** 2022-03-18

**Authors:** Isabella J. M. Niesten, Harald Merckelbach, Brechje Dandachi-FitzGerald, Ingrid Jutten-Rooijakkers, Alfons van Impelen

**Affiliations:** ^1^Clinical Psychological Science, Maastricht University, Maastricht, Netherlands; ^2^Clinical Psychology, Open University of the Netherlands, Heerlen, Netherlands

**Keywords:** clinical decision making, malingering, tunnel vision, negative predictive power, debiasing, feigning, antisocial personality features, symptom validity testing

## Abstract

Feigning (i.e., grossly exaggerating or fabricating) symptoms distorts diagnostic evaluations. Therefore, dedicated tools known as symptom validity tests (SVTs) have been developed to help clinicians differentiate feigned from genuine symptom presentations. While a deviant SVT score is an indicator of a feigned symptom presentation, a non-deviant score provides support for the hypothesis that the symptom presentation is valid. Ideally, non-deviant SVT scores should temper suspicion of feigning even in cases where the patient fits the DSM’s stereotypical yet faulty profile of the “antisocial” feigner. Across three studies, we tested whether non-deviant SVT scores, indeed, have this corrective effect. We gave psychology students (Study 1, *N* = 55) and clinical experts (Study 2, *N* = 42; Study 3, *N* = 93) a case alluding to the DSM profile of feigning. In successive steps, they received information about the case, among which non-deviant SVT outcomes. After each step, participants rated how strongly they suspected feigning and how confident they were about their judgment. Both students and experts showed suspicion rates around the midpoint of the scale (i.e., 50) and did not respond to non-deviant SVT outcomes with lowered suspicion rates. In Study 4, we educated participants (i.e., psychology students, *N* = 92) about the shortcomings of the DSM’s antisocial typology of feigning and the importance of the negative predictive power of SVTs, after which they processed the case information. Judgments remained roughly similar to those in Studies 1–3. Taken together, our findings suggest that students and experts alike have difficulties understanding that non-deviant scores on SVTs reduce the probability of feigning as a correct differential diagnosis.

## Introduction

Symptom exaggeration as seen in patients who feign their health complaints may distort diagnostic evaluations. A survey by Mittenberg et al. (2002) estimated the base rate of feigned complaints to be within the 10–30% range in criminal and civil—i.e., personal injury/disability—cases. Non-trivial rates have, however, also been reported outside of the legal domain. For instance, Dandachi-FitzGerald et al. (2011) reported a prevalence of 10–30% among psychiatric outpatients. Comparable estimates have been reported for patients with vague physical and neurological symptoms that are diagnosed with labels such as fibromyalgia and persistent mild head injury [e.g., see Stulemeijer et al. (2007) and Johnson-Greene et al. (2013)].

Unfortunately, clinical judgment is a suboptimal tool for distinguishing between valid and non-valid symptom presentations. For example, Hickling et al. (2002) sent six actors who simulated post-traumatic stress disorder (PTSD) to a treatment facility and found that clinicians initially did not detect them. Once informed about the presence of simulators among patients, clinicians correctly classified only 50% of the simulators (true positives) and misclassified 57% of genuine patients (false positives). Reviewing 12 studies into clinicians’ ability to distinguish feigned from genuine pathology, Rosen and Phillips (2004, p. 3) concluded: “When questioned about the actual occurrence of subjective symptoms, or the truthfulness of a patient’s report, the wise clinician would do well to be less than certain.” Subsequent studies, both in the clinical (Dandachi-FitzGerald et al., 2017) and the forensic (Ng et al., 2021) domain supported this conclusion.

If clinicians cannot rely on their own intuition, then when should they be suspicious about the validity of their patients’ symptoms? The Diagnostic and Statistical Manual for Mental Disorders-5 (DSM-5; American Psychiatric Association, 2013) advises practitioners to take feigning—i.e., malingering—into account when any combination of the following features arise; the patient (1) is involved in a medicolegal procedure, (2) reports subjective pathology that is not corroborated by objective findings, (3) does not cooperate fully with diagnostic procedures, and/or (4) meets criteria for an antisocial personality disorder (ASPD). The DSM’s portrayal of malingering was introduced in the 1980s and has not been revised since. Yet, it has received much criticism because it is so a-specific and vague that it applies to large groups of patients (Berry and Nelson, 2010; Ray et al., 2013; Niesten et al., 2015; Watts et al., 2016; van Impelen et al., 2017). In fact, at least half of forensic patients exhibit at least two of these features. Rogers (1990) warned that using the DSM’s typology to detect feigning inevitably leads to an unacceptable misclassification rate of genuine patients as feigners (false positives). Thus, relying on the DSM as a departure point to establish whether symptoms are valid is certainly not without risk.

Indeed, a large body of research has shown that clinicians’ diagnostic decisions are affected by (irrelevant) background information [e.g., Croskerry (2009) and Croskerry et al. (2013)]. For example, clinicians frequently rely on the patient’s history as an anchor to guide subsequent clinical evaluations. Yet, if the self-reported history is incomplete or misleading, this can result in lower diagnostic accuracy due to a failure to scale down initial impressions [see also Sibbald and Cavalcanti (2011)]. Similarly, clinicians are known to compare patients to “prototypes” or “scripts” of hypothetical patients that are easily accessible in memory (i.e., heuristics) and serve swift decision-making (Garb, 1996). Prototypes tend to be guided by clinical lore rather than empirical data and can pose a threat to diagnostic accuracy due to premature satisfaction with initial hypotheses (Elstein, 1994; Elstein and Schwarz, 2002; Galanter and Patel, 2005; Wakefield, 2012). Although the DSM is not a prototype-system given its reliance on strict criteria (Wakefield, 2012), prototypes may be reinforced by DSM’s portrayals: both originate from consensus among practitioners, which is strongly affected by clinical wisdom.

To reduce clinical judgment error, many researchers and professional organizations agree that clinicians should include Symptom Validity Tests (SVTs) when they try to rule in or out feigning in their patients (McMillan et al., 2009; Committee on Psychological Testing, 2015; Sweet et al., 2021). In the context of this paper, we use the superordinate term SVT to refer to both self-report validity tests that tap into symptom over-reporting and Performance Validity Tests (PVTs) that assess cognitive underperformance [for an overview of such tests, see Young (2014)]. An example of a widely used SVT that targets over-reporting is the *Structured Inventory of Malingered Symptomatology* [SIMS; see for an overview van Impelen et al. (2014) and Shura et al. (2021)]. The SIMS taps into symptom over-reporting by having patients respond to a list of atypical symptoms. Scores above the cutoff of 16 are suggestive of symptom exaggeration. Analogue research has shown that this cutoff has a relatively high sensitivity (i.e., 90%) and a relatively low rate of false positives (i.e., <10%) (van Impelen et al., 2014). SVTs that tap into underperformance—also known as PVTs—consist of reasoning- or memory tasks that are so easy that even young children and patients with brain damage perform relatively well on them. An example is the Amsterdam Short-Term Memory test (ASTM; Schmand et al., 1999; Schmand and Lindeboom, 2005). The ASTM relies on a forced-choice word-recognition procedure. Correct answers are summed (0–90) to obtain a total score. Scores below 85 are suggestive of underperformance. This cutoff has reasonably good sensitivity (91%) and specificity rates (i.e., false positives <12%; Schmand and Lindeboom, 2005).

For a long time, test developers referred to SVTs as *malingering* instruments. Given this focus on identification of positive cases (i.e., feigning, malingering), in other words the sensitivity of the test, clinicians may not realize that there is another outcome that is at least as important: Unremarkable SVT scores. When a patient obtains a non-deviant score on an SVT—e.g., <16 on the SIMS or >85 on the ASTM (i.e., a score in the passing range)—this provides support for a credible symptom presentation. Technically, this refers to the negative predictive power (NPP) of SVTs. As an example, with a cutoff of 16 (and a base rate of feigning ranging from 10 to 50%), the SIMS has a NPP above 0.85, indicating that the chance that an individual is *not* feigning is ≥85% if their SIMS-scores are non-deviant [see van Impelen et al. (2014)]. Similar considerations apply to the ASTM. In other words, clinicians should not only take into account deviant but also non-deviant SVT scores when evaluating symptom validity.

With these considerations in mind, the present paper explored what happens when a clinical case fits neatly with the DSM’s typology of feigning but is accompanied by non-deviant SVT scores. Are students or clinicians able to take the informational value of such scores into account and adapt their clinical judgment? Or do they stick with their initial judgment and disregard the disconfirming evidence (i.e., do they display conformation bias)? Relying on an approach by Oskamp (1965), we presented (future) experts (Studies 1, 2, and 3) with diagnostic information in a sequential manner and examined how their diagnostic judgments developed over time. Finally, we explored whether confirmation bias in diagnostic judgments regarding feigning can be tempered by providing corrective information about the DSM’s shortcomings and the importance of SVTs and their NPP (Study 4). On the basis of earlier studies [see, for a review, Richards et al. (2015)], we hypothesized that participants would show raised initial suspicion rates and that those rates would remain stable over time, despite the provision of disconfirming evidence in the form of non-deviant SVT scores. As for Study 4, we hypothesized that such rates would be sensitive to debiasing information, although we realize that studies have found that educating experts is a weak form of debiasing (Lilienfeld et al., 2009).

## Study 1

### Participants

The sample consisted of 55 graduate students who studied legal or forensic psychology at the Faculty of Psychology and Neuroscience of Maastricht University, the Netherlands. Although we did not collect data on age and sex, a fair estimate would be that most participants’ age ranged between 22 and 25 years and that the majority (±75%) were women. These estimates are based on the demographics of students typically enrolled in psychology programs [e.g., Cope et al. (2016)]. Participation in the study was not compensated. The study was approved by the standing ethical committee of the Faculty of Psychology and Neuroscience of Maastricht University.

### Measures and Procedure

Prior to a lecture, students were briefly presented with a patient case on paper.^1^ The case and a graphical overview of the procedures can be found in Supplementary Appendixes A and B, respectively. We embedded suggestions in line with the DSM’s typology of feigning into the case. More specifically, the case concerned a 55-year old asylum seeker, who said he recently started experiencing migraine-like headaches and intrusions relating to a traumatic event; he had been in the country illegally since 1995 and was able to speak the language properly, yet was currently facing the possibility of having to return to his country of origin; he had a criminal record, which included—amongst other things—being drunk in public; and he had been advised by his physician to consult a neurologist, but had never followed through on this advice. The referral question was as follows: “How valid are the symptoms of this patient?”

Students were asked to assume the role of diagnostician and to base their conclusions on the information presented to them, including the patient’s self-reports and scores on various tests. Once students had read the initial information regarding the case, they judged (1) how realistic the case was; (2) if they, at this stage, thought that they were dealing with a patient who was feigning; and (3) how confident they felt about this judgment. Answers were provided through scales ranging from 0 to 10 (e.g., 0 = not realistic at all; the chance that this individual is feigning is zero; I am not at all confident about my judgment; 10 = Very realistic case; the chance that this individual is feigning is very high; I am very certain about my judgment). Subsequently, students were provided with new information in five consecutive rounds. In round 1 (SIMS), they were given brief information regarding the SIMS and were told that the patient obtained a score of 14 (i.e., on the non-deviant, unremarkable side of the cutoff). In round 2 (Hobby), neutral information regarding the patient’s hobbies followed (i.e., the patient said he enjoyed walking the neighbor’s dogs). In round 3 (ASTM), students received basic information regarding the ASTM and were told that the patient obtained a score of 87 (i.e., again on the non-deviant side of the cutoff). In round 4 (Interview), details from a clinical interview were addressed: The patient reported that his complaints had become particularly excruciating once he found out that he may have to leave the Netherlands, that he was afraid that his symptoms were the result of a brain tumor, and that symptoms occurred approximately once or twice a week and would last all day. In round 5 (Psychometrics), psychometric details were provided, which implied that the patient reported many complaints on the Symptom Checklist-90 (SCL-90; Derogatis, 1994) and the PTSD Symptom Scale (PSS-1, Foa et al., 1993). We counter-balanced the presentation of information over the rounds to minimize order effects.^2^

After each round, students rated on 11-point scales (0–10) how likely (L) they estimated it to be that the patient was feigning and how confident (C) they were about their judgment (see above). Furthermore, they were asked to indicate how they would formulate their findings in the final report to the referee. That is, after each new piece of information they had to choose one of three conclusions, which were (1) the findings are in support of genuine pathology/provide no indication that the patient is feigning (i.e., no feigning), (2) the findings raise questions (i.e., possible feigning), or (3) the findings are suspicious/suggest feigning (i.e., feigning). As an example, when presented with the patient’s results on the SIMS, participants could choose to report: “Mr. X. scored within the normal range on the SIMS. Therefore, there is no indication that he is feigning his symptoms”, or “Mr. X. scored just below the cutoff of the SIMS, which raises questions” (i.e., possible feigning), and “Mr. X. obtained a suspicious score on the SIMS” (i.e., feigning). When analyzing these data, we collapsed the answer categories possible feigning and feigning because both convey raised suspicion that the symptoms may be invalid.

### Results and Discussion

**FIGURE 1:**
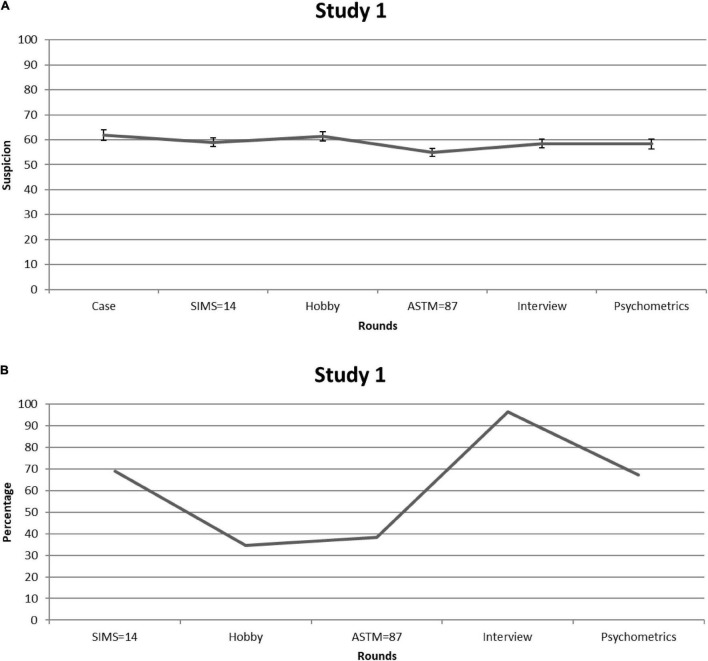
Study 1. **(A)** Students’ mean feigning x confidence ratings after each round of information. Error bars are Standard Errors of the Mean. **(B)** Percentages of students (per round) who considered mentioning (possible) feigning as a conclusion in their reports.

Students rated the case to be fairly realistic (*M* = 7.25, *SD* = 1.31). To keep the number of tests to a minimum, we averaged the likelihood and confidence ratings [([L+C]/2) × 10] and used this “suspicion” score in a repeated measures Analysis of Variance (but see Supplementary File A for likelihood and confidence ratings when examined separately). Figure 1A displays suspicion scores for each consecutive round. Given that Mauchly’s test indicated that sphericity had been violated [χ^2^_(14)_ = 30.65, *p* = 0.006], we relied on Greenhouse-Geisser corrections (ε = 0.82). Suspicion significantly fluctuated over the rounds—*F*_(4.10,221.51)_ = 3.62, *p* = 0.007, partial η^2^ = 0.063. However, the average value (i.e., *M* = 58.99, *SE* = 1.42) remained well above the center of the scale (50). *Post-hoc* analyses revealed no significant decline in suspicion rates after SIMS information relative to the other rounds (all *p*s > 0.05). Ratings in response to the patient’s score on the ASTM were significantly lower when compared with initial ratings [i.e., Case; *M* = –6.82, 95% CI (–10.61, –3.03), *p* = 0.001] and ratings provided after having read information regarding the patient’s Hobby [*M* = –6.32, 95% CI (–9.74, –2.90), *p* = 0.001], but not in comparison with the other rounds (all *p*s > 0.05, after correcting for multiple comparisons). Figure 1B shows the percentage of students who reported they would mention their suspicion in their diagnostic report. This proportion was on average 61%.

To sum up, students showed elevated suspicion from the start. More importantly, non-deviant SVT scores did not seem to have lasting corrective effects on judgment, with only the ASTM temporarily reducing suspicion in comparison with information provided during some other rounds (e.g., Hobby). That the clinical meaning of this decline may be trivial becomes apparent when looking at the percentage of students who would consider mentioning (possible) feigning in their final report: Over half of the students chose this option despite having been presented with two non-deviant SVT scores. This finding underlines that graduate psychology students—who are enrolled in legal/forensic psychology programs—do not consider the negative predictive power of SVTs when confronted with a case vignette that at first sight fits with the typical profile of a feigning patient. In Study 2, we explored the issue among experts.

## Study 2

### Participants

We used the snowball-method to recruit experts. In total, 42 psychologists and psychiatrists participated in the study. Of these experts, 19 (9 women) worked as forensic psychologists, and 23 (16 women) worked as clinical psychologists in a non-forensic setting. The majority of experts had been working in the field for over 10 years (*M* = 12.01, *SD* = 10.33). No compensation was given for study participation. The study was approved by the standing ethical committee of the Faculty of Psychology and Neuroscience of Maastricht University.

### Measures and Procedure

Experts were presented with the asylum seeker case of Study 1 via e-mail or on paper and followed the same procedure. That is, in successive steps they received information about the patient. After reading the initial case information, they judged (1) how realistic they found the case, and (2) how likely they thought it to be that the patient was feigning, and (3) how confident they were in this judgment (i.e., on scales from 0 to 10). After each of the subsequent five rounds, experts rated on 11-point scales how likely (L) they estimated it to be that the patient was feigning and how confident (C) they were about their judgment. Furthermore, they indicated how they would formulate their findings in the final report to the referee (coded as supporting a conclusion of genuine pathology, possible feigning, or feigning; see Study 1).

Like in Study 1, we averaged likelihood and confidence ratings [([L+C]/2) × 10] and employed this score in a 2 (groups: forensic versus clinical experts) × 6 (background information; rounds 1–5) ANOVA with repeated measures on the last factor (see Supplementary File A for Likelihood and Confidence ratings when examined separately). Additionally, by means of χ^2^-tests, we looked at the percentage of experts who would mention suspicion of feigning in their diagnostic report and how this fluctuated over the rounds.

### Results and Discussion

**FIGURE 2:**
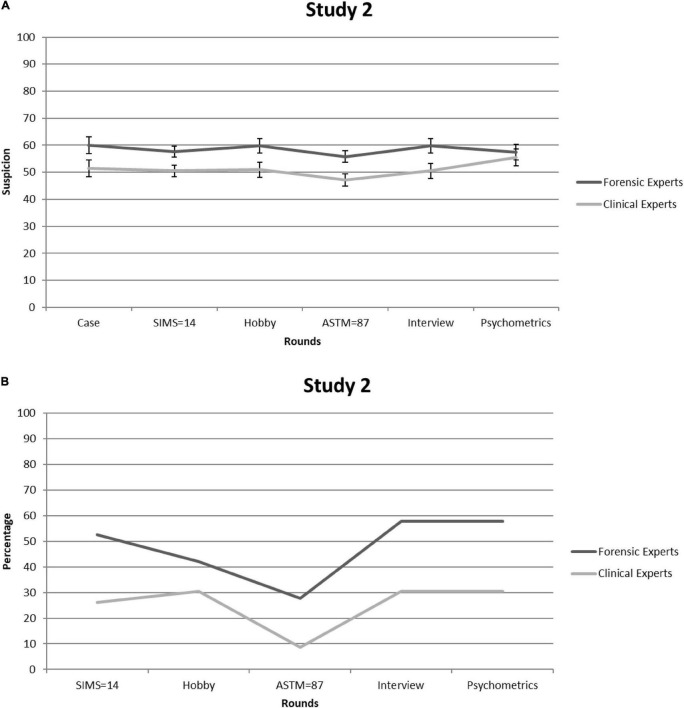
Study 2. **(A)** Forensic and clinical experts’ mean feigning x confidence ratings after each round. Error bars are Standard Errors of the Mean. **(B)** Percentages of forensic and clinical experts (per round) who considered mentioning (possible) feigning as a conclusion in their reports.

Both groups rated the case as highly realistic—forensic experts: 8.11 (*SD* = 0.94); clinical experts: 7.57 (*SD* = 1.12)—and there were no significant differences in these ratings between groups [*t*_(40)_ = –1.6, *p* = 0.10]. Figure 2A shows suspicion scores [=([L + C]/2) × 10] for each consecutive round. Given that Mauchly’s test indicated that sphericity had been violated [χ^2^_(14)_ = 50.66, *p* < 0.001], we relied on Greenhouse-Geisser corrections (ε = 0.69). Three aspects of the observed pattern are of particular interest. First, suspicion was—regardless of time point—stronger among forensic than clinical experts: 58.38 (*SE* = 2.12) versus 51.00 (*SE* = 2.01), *F*_(1,38)_ = 6.39, *p* = 0.016, partial η^2^ = 0.14. This may reflect the fact that base rates of feigning are higher in forensic than in general clinical settings (Niesten et al., 2015). Thus, forensic experts might be more familiar with the literature—including the DSM-5—on this topic. Second, suspicion barely fluctuated over the rounds—*F*_(3.46,131.39)_ = 1.76, *p* = 0.15, partial η^2^ = 0.044—and circled around the center of the scale (i.e., 50). This suggests that experts were not very sensitive to the informational value of the SVT scores. Third, forensic experts and clinical experts did not differ in this regard, but were equally insensitive to non-deviant SVT outcomes: *F*_(3.46,131.39)_ = 1.10, *p* = 0.36, partial η^2^ = 0.028.

Figure 2B shows the percentage of experts who would mention suspicions of feigning in their diagnostic report. This time, no significant differences arose between the two expert groups [all χ^2^s_(1)_ < 3.2, all *p*s > 0.07]. Although the proportion of experts who considered mentioning suspicion of feigning was lower than that of students in Study 1, it was nevertheless substantial, with the averages over rounds being 25 and 48% for clinical and forensic experts, respectively.

Our data demonstrate that experts do not seem to benefit from the informational value of non-deviating SVT scores. Admittedly, our sample of experts was small and they all received the same case. It is possible that a larger sample and other types of cases would yield different results. Therefore, in Study 3, we recruited a larger sample of clinical experts and provided one group with the asylum seeker case and the other with a new case (see below).

## Study 3

### Participants

We asked clinical psychologists at a Dutch clinical psychology conference to participate in the study prior to attending a lecture on symptom validity. In total, ninety-three clinical experts^3^ completed the study. There was no reward for participation. The study was approved by the standing ethical committee of the Faculty of Psychology and Neuroscience of Maastricht University.

### Measures and Procedure

The procedure was similar to that of Study 1 and 2, except that some participants received the original case (*n* = 57) and some received another case (*n* = 36). Briefly, the new case concerned a 55-year old patient who was the victim of a car accident 15 months earlier (i.e., rear-end collision with a truck at a traffic light). He was taken to the hospital, stayed there several days, and since then had been experiencing various complaints (e.g., poor concentration, frequent nausea, heightened irritation, inability to cope with several tasks at the same time). He took up sick leave from work, occasionally visited the doctor/neurologist, and was involved in a litigation procedure against the truck driver. Before the accident occurred, the patient had been through a tough divorce; his ex-wife accused him of having a personality disorder. Furthermore, he had a longstanding reputation for not paying his apartment bills, resulting in several eviction warnings from the housing corporation. Immediately on entering the examination room of the psychologist, he remarks that he does not feel like completing a test battery again. The referral question was as follows: “Is it likely that the patient’s symptoms are part of a post-traumatic stress disorder?.”

Experts judged (1) how realistic they found the case, and for each sequential step they rated (2) if they thought the patient was feigning, and (3) how confident they were in this judgment (both on scales from 0 to 10). In steps, they were informed that the patient (1) obtained a SIMS score of 14 (i.e., on the non-deviant side of the cutoff), (2) enjoyed walking the neighbor’s dogs (i.e., Hobby), (3) obtained a score of 87 on the ASTM (i.e., again on the non-deviant side), (4) reported that his complaints had become particularly excruciating since the accident, that he worried he would never again be able to work again, and that the complaints occurred approximately once or twice a week and would last all day (i.e., Interview), and (5) obtained a clinically raised score on the Symptom Checklist-90 (SCL-90; Derogatis, 1994) and the PTSD Symptom Scale (PSS-1; Foa et al., 1993) (i.e., Psychometric information). After each step, experts also indicated how they would formulate their findings in the final report to the referee (see Studies 1 and 2).

We averaged their likelihood and confidence ratings [([L+C]/2) × 10] and used this score in a 2 (case) × 6 (background information; rounds 1–5) ANOVA with repeated measures on the last factor (see Supplementary File A for likelihood and confidence ratings when examined separately). Additionally, we examined the percentage of experts who said that they would mention suspicion of feigning in their diagnostic report and how this fluctuated over the consecutive rounds.

### Results and Discussion

Both cases were rated as realistic – asylum seeker: 7.36 (*SD* = 1.63); car accident: 7.56 (*SD* = 1.00), and these ratings did not significantly differ between groups [Welch *t*_(89.83)_ = –0.72, *p* = 0.47]. There was an interaction between case and rounds, *F*_(4.23,342.28)_ = 2.68, *p* = 0.029, partial η^2^ = 0.032. Follow-up ANOVAs showed that suspicion scores did not statistically differ between cases per round (all *p*s > 0.05). However, there was a significant effect of round on suspicion scores in the asylum seeker case [*F*_(5.00,230.00)_ = 3.21, *p* = 0.008, partial η^2^ = 0.065] and not in the car accident case [*F*_(3.03,105.92)_ = 2.01, *p* = 0.12, partial η^2^ = 0.054]. Still, pairwise comparisons showed no significant differences between time points after correction for multiple comparisons (i.e., Tukey). Furthermore, percentages of clinicians who would mention (possible) feigning in their report after each round did not differ significantly between the two cases [all χ^2^s_(2)_ < 5.58, all *p*s > 0.061].

**FIGURE 3:**
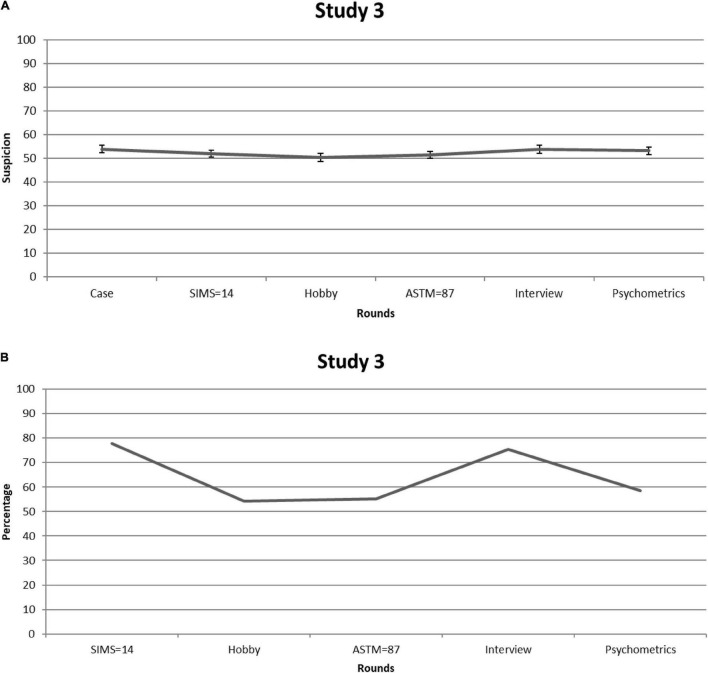
Study 3. **(A)** Clinical experts’ mean feigning x confidence ratings after each round. Error bars are Standard Errors of the Mean. **(B)** Percentages of clinical experts who (per round) considered mentioning (possible) feigning as a conclusion in their reports.

Given that differences between cases were negligible, we collapsed the data. Figure 3A shows suspicion of feigning [=([L + C]/2) × 10] over each consecutive round. Because Mauchly’s test showed that sphericity had been violated [χ^2^_(14)_ = 31.03, *p* = 0.006], we relied on Greenhouse-Geisser corrections (ε = 0.86). Suspicion significantly fluctuated over the rounds—*F*_(4.32,353.93)_ = 2.48, *p* = 0.04, partial η^2^ = 0.029—but, on average, circled around the center of the scale (i.e., *M* = 52.51, *SE* = 1.33), suggesting that experts were not very sensitive to the informational value of the SVT scores. Indeed, over time, only minor changes in ratings appeared (i.e., the largest was 3.5 scale points) and none of the changes in clinicians’ scores in response to the SIMS and ASTM were significant (all *p*s > 0.05).

Figure 3B shows the percentage of experts who said they would mention suspicion of feigning in the diagnostic report. The overall mean percentage of clinicians who would report suspicion of feigning was 64%. Noteworthy, however, clinicians who opted for (possible) feigning frequently provided elaborations for choosing this option—i.e., in contrast to the previous studies. Most of the times, these elaborations also reflected doubts about whether or not the patient was feigning. Thus, even though these clinicians opted for (possible) feigning as a differential diagnosis in their report, it seems that they were not dogmatic about their conclusions.

The findings of Study 3 largely reiterate the findings of Study 1 and 2; that is, clinicians had elevated initial suspicion rates that barely changed when exposed to non-deviant SVT scores. They were also inclined to report feigning in their report, though a sizeable number of clinicians seemed to feel uncertain about this conclusion. This observation underlines the possibility that they had difficulties understanding the informational value of non-deviant SVT scores. This suggests the need for tools to guard clinicians against drawing wrong diagnostic conclusions regarding symptom validity.

## Study 4

### Participants and Procedure

In Study 4, we explored whether a brief educational intervention would improve judgments of feigning. Ninety-two graduate students in legal or forensic psychology at the Faculty of Psychology and Neuroscience of Maastricht University judged a case (i.e., asylum-seeker) after receiving a 2-h lecture on the limitations of the DSM as well as explanations of SVTs and NPP. Importantly, we included data from the 55 students in Study 1 because they had judged the case both prior (i.e., results discussed under Study 1) and after this lecture (i.e., results discussed below; see also Supplementary File B) and complemented their data with a later cohort of 37 students who had judged the case once, after receiving the same lecture.^4^ We did not collect data on mean age and sex. However, a fair estimate would be that the majority of participants were aged between 22 and 25 years and that most (±75%) were women [e.g., see Cope et al. (2016)]. There was no reward for participation. The study was approved by the standing ethical committee of the Faculty of Psychology and Neuroscience of Maastricht University.

### Results and Discussion

Students rated the case to be fairly realistic (*M* = 7.32, *SD* = 1.35). To keep the number of tests to a minimum, we averaged the likelihood and confidence ratings [([L+C]/2) × 10] and used this “suspicion” score in a repeated measures ANOVA (see Supplementary File A for likelihood and confidence ratings when examined separately).

**FIGURE 4:**
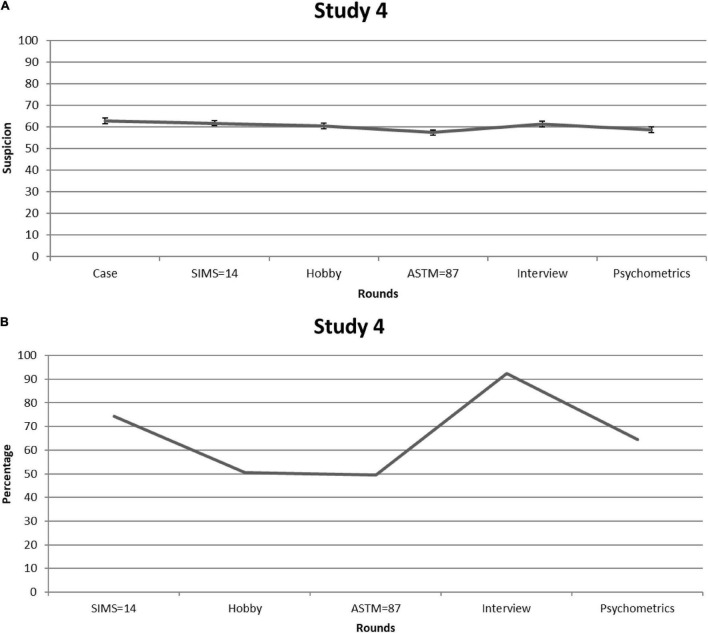
Study 4. **(A)** Mean feigning x confidence ratings after each round of students who received information about the diagnostic and statistical manual for mental disorders (DSM’s) shortcomings and the importance of negative predictive power (NPP). Error bars are Standard Errors of the Mean. **(B)** Percentages of students (per round) who considered mentioning (possible) feigning as a conclusion in their reports.

Figure 4A shows suspicion for feigning [=([L + C]/2) × 10] for each consecutive round. Given that Mauchly’s test indicated that sphericity had been violated [χ^2^_(14)_ = 33.96, *p* = 0.002], we relied on Greenhouse-Geisser corrections (ε = 0.86). Again, suspicion significantly fluctuated over the rounds—*F*_(4.30,386.51)_ = 6.53, *p* < 0.001, partial η^2^ = 0.068—but on average hovered above the center of the scale (i.e., *M* = 60.42, *SE* = 1.07). Participants’ suspicion scores after the SIMS were significantly *higher* than after the ASTM [*M* = 4.29, CI (2.42, 6.15), *p* < .001], and after psychometric information [*M* = 3.08, 95% CI (1.07, 5.09), *p* = 0.003; all other rounds *p* > 0.05]. Ratings in response to the patient’s score on the ASTM were significantly lower, not only compared with the SIMS, but also compared with the other rounds [i.e., Case *M* = –5.44, 95% CI (–7.88, –3.00), *p* < 0.001; Hobby *M* = –3.08, 95% CI (–5.02, –1.13), *p* = 0.002; Interview *M* = –4.01, 95% CI (–5.95, –2.07), *p* < 0.001], except for psychometric information (*p* = 0.218).

The pattern of findings is comparable to that of Study 1: SVTs—the ASTM in particular—may temporarily reduce suspicion in a statistically significant fashion, but in clinical practice the effect seems subtle. This is further demonstrated by Figure 4B: The proportion of students who would raise their suspicions of feigning was roughly the same at the beginning and the end of the steps. That is, on average 66% stated that they would mention (possible) feigning in their final report.

Thus, even when presented with further corrective information, initial suspicion rates were relatively high and barely changed over time. Although SVTs did correct judgment, this effect was not impressive and failed to last over the subsequent rounds (as was the case in Study 1). More worrisome, slightly more than half of the students considered mentioning (possible) feigning in their final report, demonstrating that students did not become more cautious in their conclusions when provided with further corrective information.

## General Discussion

We assume that clinicians administer SVTs to their patients when they have reasons to do so [e.g., Dandachi-FitzGerald et al. (2013)]. We also assume that clinicians infer these reasons from one of the most influential sources in their files, namely the DSM and what it says about when to expect malingering. What happens when clinicians are presented with cases that fit the DSM’s malingering section? Our findings (Study 1–3) suggest that they will use it as an anchor, creating room for tunnel vision during diagnostic decision-making. Indeed, our participants’ scores were raised from the start and remained roughly similar over subsequent rounds. We also obtained tentative evidence that the inclination to stick to an initial impression of feigning may not be easily amended (Study 4): Future experts who had first been provided with a lecture that included debiasing information still showed as high a propensity to anchor their judgment toward feigning as did those who had not been provided with this information prior to completing the case.

To our knowledge, we are the first to provide data on an issue that has been largely overlooked in symptom validity research: How do experts weigh non-deviant scores on SVTs? Do they incorporate the disconfirming evidence that such scores provide into their judgments or will their initial—potentially erroneous—impression prevail? The latter seems to be the case. While non-deviant scores on the SIMS and the ASTM occasionally went hand in hand with a reduction in suspicion rates (i.e., in Study 1 and 4), decreases were modest in size and therefore of questionable clinical impact. One could argue that our participants displayed relatively low degrees of suspicion given that their values fluctuated around the midpoint of the scale (i.e., 50). At first sight, this observation may be interpreted as general diagnostic cautiousness; that is, experts are already careful in their judgments and therefore non-deviant scores on SVTs do not have additional value. While this is a more optimistic interpretation, it does not square with the observation that across studies a substantial number of participants considered mentioning (possible) feigning in their report. Although experts in Study 3 oftentimes elaborated on their choice for (possible) feigning in ways that implied uncertainty about the selected diagnostic conclusion, this finding once more underscores that there may be considerable confusion among practitioners when it comes to interpreting non-deviant SVT results. Such confusion may have a detrimental impact on diagnostic accuracy in clinical practice.

There are additional considerations in relation to our findings. First, the DSM-5 uses a poorly demarcated typology of feigning that has a pejorative tone (Berry and Nelson, 2010; Niesten et al., 2015). This provides a fruitful ground for tunnel vision, in which the first diagnostic impression is not corrected in the face of subsequently obtained disconfirming evidence (Oskamp, 1965; Berner and Graber, 2008). Second, our findings are reminiscent of more fundamental research that has provided evidence of asymmetrical flexibility in the development of decisions; people are more flexible in accepting than rejecting a hypothesis (Gilbert, 1991). This phenomenon also applies to the diagnostic hypotheses of medical and psychological experts (Berner and Graber, 2008) as well as psychotherapists and psychiatrists (Crumlish and Kelly, 2009). For example, Spaanjaars et al. (2015) found that (moderately experienced) clinicians used a referral letter suggestive of depressive complaints as an anchor for both a preliminary and final diagnosis of depressive disorder.

With respect to feigning, asymmetrical flexibility is fostered in two ways. First, the DSM-5 gives the impression that feigning is categorical in nature: The (pseudo)patient is feigning *or* the patient is honest. Thus, diagnosticians are required to weigh a series of probabilistic indications—the results of tests and interviews—and to translate them into a categorical decision. People—including experts—are not good at this and often show a tendency to lower their *beyond-reasonable-doubt* criterion (Magnussen et al., 2014). As a result, minimal indications—e.g., vague pointers in the background information of a case—may cause experts to feel tempted to conclude that a patient is feigning. Yet, other indications—e.g., non-deviant SVT results—are not used as falsification of this conclusion. A second source of asymmetrical flexibility might be the way researchers who develop SVTs tend to write about their instruments. Their meta-analyses and manuals devote attention to sensitivity and the low false-positive rates of their tests [see for an illustration: Sollman and Berry (2011)]. That is, SVTs are usually presented as tools to detect those who feign, but the NPP of SVTs is at least as important [see for a more detailed account on NPP, Rosenfeld et al. (2000)].

Briefly, if the base rate of feigning is set at 30%, the degree to which the below SVT cutoff scores used in our case vignettes are representative of a credible symptom presentation is ±0.98 for the SIMS and ±0.97 for the ASTM, with a false negative rate of ±2% and ±3%, respectively. Given that scores were within the normal range on two SVTs that are reasonably independent of each other, the likelihood that this patient is a false negative—i.e., the patient is feigning, but is classified as honest—becomes even lower, namely ±0.06%. While such a pattern of scores should considerably temper experts’ suspicion rates, our results show that non-deviant SVT scores do not have this corrective effect.

Admittedly, our studies have several limitations. First, we did not contrast our case to a case devoid of references to the DSM’s typology of feigning. If initial suspicion rates for such a case are found to be substantially lower, this would provide further support for our assertions regarding the potential anchoring effects of DSM stereotype on clinical decision making. We also did not include a condition in which the patient obtained deviant SVT outcomes whilst not fitting the DSM’s typology. Evidently, it would be informative to see whether or not deviant SVT scores would be disregarded in such a case. Second, our cases were brief and consisted solely of written information. Participants were not given the opportunity to formulate their own questions, collect collateral information, access scientific literature (e.g., about base rate estimates) and test-specific classification accuracy data. They were also not given the opportunity to select their own tests. It is possible that had they themselves chosen to include SVTs in the test battery or been allowed to make a selection out of a list of SVTs, they would be less likely to disregard the (non-deviant) outcomes. Furthermore, most of our participants only judged one case, which may limit the extent to which our findings can be generalized to the broad variety of cases encountered in clinical practice. However, we presented a subgroup of our experts with another case (see Study 3) and obtained roughly similar results, highlighting the potential generalizability of the effect. Third, we relied on two SVTs. Presenting other—and perhaps an additional number of—SVTs may have larger corrective power. Finally, to evaluate our debiasing intervention, we combined data from participants in Study 1 and 4. This may have introduced a confounding element because participants in Study 1 had been repeatedly exposed to the same case, which could have affected their ratings (e.g., due to a preference for consistency). Future studies ideally present participants with two different cases in a counter-balanced order, and provide half of participants with debiasing information and the other half with neutral information that should not in any way affect the ratings. This way, the actual effect (or lack of effect) of debiasing could be more thoroughly studied. Noteworthy though, solely providing debiasing information is considered a rather weak form of debiasing [see Lilienfeld et al. (2009)]. Debiasing strategies that require active engagement from participants may be more successful at achieving diagnostic accuracy. Such strategies may, for instance, require participants to actively produce opposite explanations for the diagnostic findings at hand or, in the specific case of feigning, require participants to actively consider the NPP (and PPP) for different base rates before drawing a final diagnostic conclusion.

The topic is important: Dandachi-FitzGerald et al. (2017) reported that a combination of initial suspicion of feigning and non-deviant SVT scores occurs frequently among hospital patients referred to a neuropsychologist. When these researchers asked neuropsychologists to predict SVT performance and compared their predictions with actual SVT outcome data, they found that of the 51 patients who had been predicted to have problematic symptom validity, as many as 35 (68%) had, in fact, passed both SVTs. The degree to which the DSM’s profile of feigning drove these clinicians to incorrect classification is unknown. Yet it may be considerable: As said before, clinicians rely on prototypes—or illness scripts—when they evaluate patients [e.g., Garb (1996, 2005)]. This can cause anchoring toward a hypothesis that has low diagnostic accuracy, particularly when there seems to be no viable substitute script.

Diagnostic and Statistical Manual for Mental Disorders typology of feigning provides a strong and intuitively appealing script. Furthermore, while some clinicians may be aware of its low predictive utility, a competing script that readily places somewhat ambiguous (i.e., below, but close to the cutoff) SVT scores into context may not readily be available. As a result, they may fail to select the most probable hypothesis (i.e., the patient likely presents with genuine symptoms despite an antisocial background). To guard clinicians against this issue, developers of SVTs could more extensively stress the meaning of NPP in their manuals and meta-analyses. Furthermore, given that Study 4 suggests that making individuals aware of bias and providing them with corrective information may not be sufficient, it would be worthwhile to teach clinicians techniques that have been shown to be promising in overriding cognitive biases in other areas of (clinical) decision-making. Such techniques may include considering the opposite (or all alternative scenarios) and delayed decision-making [for an overview of strategies, see, e.g., Lilienfeld et al. (2009)].

Clinicians often make decisions under complex and uncertain conditions (Elstein, 1994; Galanter and Patel, 2005) and the assessment of symptom validity is no exception. Future research should disentangle the decisional steps that clinicians take when issues regarding symptom validity arise to understand how these experts arrive at their diagnostic conclusions. How do they explore information to test their initial hypothesis, and does this affect their final judgment? Clearly, diagnostic decisions may not only cause diagnostic errors but also affect subsequent high-stake decisions. For instance, Mendel et al. (2011) presented psychiatrists with a case and led them to opt for a wrong initial diagnosis of major depressive episode (instead of Alzheimer’s disease). Next, they were shown 12 items, of which six alluded to the correct and six to the incorrect diagnosis, and asked to indicate the items for which they would like to obtain more information. The researchers noted that psychiatrists used a confirmatory (13%), disconfirmatory (43%), or balanced (44%) search strategy. Of those using a confirmatory approach, only 30% came to the correct diagnosis (compared with 73 and 53% of those who employed a disconfirmatory or balanced strategy, respectively), and all psychiatrists who had made an incorrect final diagnosis proposed inappropriate treatments that could have far-reaching implications (i.e., they prescribed antidepressants instead of medication for Alzheimer’s disease).

Incorrect classification of a patient’s symptoms as feigned may evidently result in a similar chain of fatal decisions [e.g., Witztum et al. (1996)]. Therefore, it could be a fruitful endeavor to more directly scrutinize clinical decision-making in studies addressing symptom validity assessment [e.g., like in Mendel et al. (2011)]. Such research may help elucidate the forces that underlie clinicians’ proclivity to stick to their initial impressions and aid in refining clinical training and practice. Indeed, once we have gained more understanding of how clinicians come to their diagnostic conclusions regarding symptom validity, this information can be harnessed to guard against sources of bias such as that conveyed by the DSM’s typology of feigning.

## Data Availability Statement

The raw data supporting the conclusions of this article will be made available by the authors, without undue reservation.

## Ethics Statement

The studies involving human participants were reviewed and approved by Ethical Research Committee of the Faculty of Psychology and Neuroscience, Maastricht University. Written informed consent for participation was not required for this study in accordance with the national legislation and the institutional requirements.

## Author Contributions

HM obtained the ethical approval. BD-F, IJ-R, and IN collected the data. IN, HM, and IJ-R performed the data processing and analyses. IN wrote the first draft of the manuscript. All authors contributed to the conception of the research and critical feedback, aided in writing and finalizing the manuscript, and approved the manuscript before submission.

## Conflict of Interest

The authors declare that the research was conducted in the absence of any commercial or financial relationships that could be construed as a potential conflict of interest.

## Publisher’s Note

All claims expressed in this article are solely those of the authors and do not necessarily represent those of their affiliated organizations, or those of the publisher, the editors and the reviewers. Any product that may be evaluated in this article, or claim that may be made by its manufacturer, is not guaranteed or endorsed by the publisher.

## Footnotes

1 Students received the case a second time after the lecture, which contained debiasing information. We combined these data with those of an additional student sample that received debiasing information prior to judging the case. The findings are reported under Study 4.

2 Aside from the order described (i.e., SIMS, Hobby, ASTM, Interview, and Psychometrics; *n* = 14), students received the information in one of the following orders: Hobby, SIMS, Psychometrics, Interview, and ASTM (*n* = 13); Interview, ASTM, SIMS, Hobby, and Psychometrics (*n* = 14); and Psychometrics, ASTM, Interview, Hobby, and SIMS (*n* = 14).

3 Ten experts had missing data for some of the time points and were therefore not included in the repeated measures ANOVA.

4 Aside from one of the samples rating the case twice (i.e., before and after a lecture), participants followed the same procedure. Importantly, participants did not significantly differ in their ratings of the case [*t*_(49.64)_ = 1.37, *p* = 0.18]. Furthermore, in terms of their suspicion rates, there was no interaction between rounds and group [*F*_(4.28,381.06)_ = 0.57, *p* = 0.70, partial η^2^ = 0.006] and no a main effect of group [*F*_(1.00,89.00)_ = 0.47, *p* = 0.50, partial η^2^ = 0.005]. Neither did percentages of participants who would mention feigning differ between Study 1 and Study 4 [all χ^2^s_(1)_ < 2.55, all *p*s > 0.11].
