# Supplementary material for: Experts’ Failure to Consider the Negative Predictive Power of Symptom Validity Tests
Source: Front Psychol. 2022 Mar 18;13:789762. doi: 10.3389/fpsyg.2022.789762 (PMC8971289; doi:10.3389/fpsyg.2022.789762)
Supplement: Supplementary file 1 [file Table_1.docx]

**Supplementary file A**

Mean (SE) for likelihood and confidence levels separately per study.

|  | | Initial information | SIMS | Hobby | ASTM | Interview | Psychometrics |
| --- | --- | --- | --- | --- | --- | --- | --- |
| Study 1 | Likelihood | 70.00 (1.82) | 60.00 (2.42)* | 64.46 (2.48) | 54.00 (2.60)** | 63.46 (1.91)* | 57.82 (3.08)* |
|  | Confidence | 53.64 (3.16) | 58.00 (2.27) | 58.18 (2.62) | 56.00 (2.23) | 53.55 (2.44) | 58.73 (2.71) |
| Study 2 | Likelihood | 63.00 (2.38) | 52.00 (2.51)* | 56.25 (2.65) | 45.25 (3.24)* | 51.25 (3.58) | 51.25 (3.48) |
|  | Condfidence | 48.05 (3.32) | 55.85 (2.59) | 54.15 (2.84) | 57.56 (2.77) | 58.78 (2.21)* | 61.71 (2.15)* |
| Study 3 | Likelihood | 60.70 (1.90) | 52.33 (2.08)* | 49.30 (2.21)** | 44.88 (2.27)** | 50.99 (2.55)* | 48.72 (2.45)** |
|  | Confidence | 47.83 (2.23) | 52.05 (2.06)* | 52.41 (2.06)* | 58.80 (2.03)** | 57.35 (2.01)** | 58.19 (1.99)** |
| Study 4 | Likelihood | 68.37 (1.42) | 61.85 (1.70)* | 63.37 (1.78)* | 55.33 (1.74)** | 64.13 (1.67) | 59.35 (1.74)** |
|  | Confidence | 57.36 (2.10) | 61.54 (1.47) | 57.47 (1.71) | 59.34 (1.77) | 58.79 (1.67) | 57.91 (1.84) |

*Note*. Reported significance levels pertain to rounds when compared to Initial information (i.e., Round 1).

* = *p* < .05
** = *p* < .001
