# Supplementary material for: Experts’ Failure to Consider the Negative Predictive Power of Symptom Validity Tests
Source: Front Psychol. 2022 Mar 18;13:789762. doi: 10.3389/fpsyg.2022.789762 (PMC8971289; doi:10.3389/fpsyg.2022.789762)
Supplement: Supplementary file 2 [file Table_2.docx]

**Supplementary File B**

Participants in Study 1 (*N* = 55 students) completed the case both prior and after receiving the debiasing intervention. To test whether their mean suspicion ratings significantly differed between the two testing occasions we conducted repeated measures ANOVAs for each of the rounds. Mean scores per round before and after debiasing are reported in Table 1. As can be seen, scores tended to be *higher* rather than lower after debiasing, but none of these differences were significant (all *F*s (1, 54) < 2.6, all *p*s > .11).

*Table 1.* Mean (SE) suspicion scores prior and after debiasing per round.

|  | Before debiasing | After debiasing | *F* (1, 54) | *p* |
| --- | --- | --- | --- | --- |
| Initial information | 61.82 (2.09) | 63.09 (1.84) | 0.19 | .66 |
| SIMS | 59.00 (1.73) | 62.46 (1.62) | 2.03 | .16 |
| Hobby | 61.32 (1.90) | 61.46 (1.80) | 0.00 | .96 |
| ASTM | 55.00 (1.59) | 57.46 (1.62) | 1.01 | .32 |
| Interview | 58.50 (1.76) | 62.73 (1.84) | 2.55 | .12 |
| Psychometrics | 58.27(1.96) | 58.91 (1.84) | 0.06 | .81 |
